# Self-Worth as a Mediator and Moderator Between Teacher-Student Relationships and Student Engagement in Rural Schools

**DOI:** 10.3389/fpsyg.2021.777937

**Published:** 2022-01-25

**Authors:** Jiali Huang, Guoyuan Sang, Tzuyang Chao

**Affiliations:** ^1^Institute of Teacher Education, Beijing Normal University, Beijing, China; ^2^Academy of Plateau Science and Sustainability, Qinghai Normal University, Xining, China; ^3^Graduate Institute of Learning and Instruction, National Central University, Zhongli, Taiwan

**Keywords:** self-worth, teacher-student relationships, student engagement, rural schools, student learning

## Abstract

This study examined how self-worth of students mediated and moderated their perceived positive teacher-student relationships and student engagement among middle-school students from rural China. Eighth graders (*N* = 838) completed surveys measuring their perceived relationships with teachers, their self-worth, and engagement. Statistical analyses revealed significant correlations among all three variables, with the strongest being between teacher-student relationships and student engagement. The structural equation modeling indicated that self-worth partially mediated the effect of teacher-student relationships on student engagement; however, positive teacher-student relationships were a stronger predictor. Multigroup analyses identified self-worth as a moderator, whereby students with lower self-worth were more reliant on positive teacher-student relationships to enhance their engagement. This study provides insights into how self-worth of students and their perceived positive teacher-student relationships influence their academic engagement in disadvantaged rural areas of China.

## Introduction

According to Ryan et al. (2013) and Skinner et al. (2009), engagement of students in learning often decreases during the transition to middle school. Students who live in impoverished rural regions are both geographically and academically distant from their urban counterparts, and they often face greater learning difficulties, higher dropout rates, and fewer higher education and career opportunities (Liu, 2019; Verkuyten et al., 2019). Studies in China have consistently identified significant gaps in the learning ability and higher education access between rural and urban students (Zhao et al., 2017). Recently, in the context of a national poverty alleviation strategy, China has strictly controlled dropouts in compulsory education. However, in a survey with seventh to ninth graders in Southwest China, Yue et al. (2016) reported that 9.4% (91 of 966) of students experienced dropping out, although they returned to campus mainly because they were persuaded by families and schools; more seriously, 49% (474 of 966) had thought of dropping out from school. Students in rural areas are usually labeled with a weak academic ability, insufficient learning motivation, and limited achievement (Liu, 2019; Verkuyten et al., 2019). In this respect, in the context of collective stereotypes and representation, they might feel devalued in the eyes of others and internalize these negative stereotypes, develop low self-evaluation, and, in turn, decrease their self-worth and engagement in learning (Crocker, 1999; Crocker and Wolfe, 2001; Brey and Pauker, 2019).

Being widely identified as a key facilitator of academic achievement (Skinner et al., 2009; Khine, 2016), student engagement is a psychological investment whereby students commit themselves to “learning, understanding, or mastering” knowledge and skills to proactively complete academic tasks (Newmann et al., 1992, p. 12). A body of research has indicated that a stronger student engagement leads to higher student achievement, as well as lower levels of student dissatisfaction and dropout rates (e.g., Quin, 2017; Lei et al., 2018). Facilitating student engagement has also been revealed to reduce or counteract the negative impact of sociodemographic predictors on student performance (Sinclair et al., 2003).

Drawing on the self-system model of motivational development (SSMMD) based on self-determination theory (SDT), as a function of individual characteristics of students, student engagement is deeply influenced by contextual factors through self-perception (Skinner et al., 2009). In particular, teacher-related elements (e.g., the style of classroom management) are definitely recognized as contextual factors influencing the engagement of students (Skinner and Pitzer, 2012; Jang et al., 2016; Pan et al., 2017; Wang and Zhang, 2020). Studies have identified that teacher-student relationships impact student engagement (e.g., Xuan et al., 2019): positive teacher-student relationships promote the disadvantaged engagement of students, whereas negative relationships often lead to lower academic engagement (e.g., Hughes and Cao, 2018).

Referring to the level of self-evaluation (Crocker and Wolfe, 2001; Hibbert, 2013), self-worth may influence engagement of students, especially in rural disadvantaged areas. Studies have indicated that self-worth is strongly influenced by members of the social network of an individual, including parents, teachers, and peers, and self-worth of students is often correlated with their performance, the quality of their relationships with teachers, and the evaluation from teachers (Crocker and Wolfe, 2001; Crocker and Luhtanen, 2003; Horberg and Chen, 2010; Ryan et al., 2013; Lavy and Naama-Ghanayim, 2020). As such, the stereotypical expectations of teachers toward the backgrounds of students and corresponding evaluations of their behavior or achievement might negatively influence the teacher-student interaction (Glock, 2016; Martin and Collie, 2019; Whitaker, 2020). Moreover, researchers have confirmed that positive self-perception and high self-worth promote student engagement, and the effect is particularly strong for students with low academic performance (e.g., Skinner et al., 2008; Lei et al., 2018).

Positive interpersonal relationships may ameliorate the geographic disadvantage on student learning; however, the effect of self-worth in this interaction remains unclear. Specifically, few studies have examined the effect of self-worth on the relation between teacher-student relationships and the engagement of students, particularly in rural contexts. Therefore, this study aimed to examine whether teacher-student relationships could directly enhance rural student engagement by bolstering the self-worth of students in rural schools in China.

## Theoretical Background and Hypotheses

### Student Engagement

Student engagement has been defined as a measure of “psychological investment in and effort directed toward learning, understanding, or mastering the knowledge, skills, or crafts of academic work” (Newmann et al., 1992, p. 12) and as participation in the effective practices, which leads to desired and measurable outcomes (Kuh et al., 2007). Previous studies have generally categorized student engagement into cognitive engagement (e.g., metacognitive strategies), affective engagement (e.g., curiosity, interest, and enthusiasm), and behavioral engagement (e.g., participatory discussion) (Fredricks et al., 2004, 2005; Wang and Holcombe, 2010). However, some studies have articulated a fourth style – engagement-agentic engagement (e.g., autonomy) – that spotlights the intentional and proactive motivational contribution of students to learning flow (Reeve, 2012; Sökmen, 2021). Student engagement is reflected through proactive cognitive, emotional, and behavioral participation in school activities; conversely, less engaged students might exhibit learning behaviors such as passiveness, refusal to participate, and frustration (Skinner et al., 2008).

Self-system model of motivational development identifies a model of internal and external dynamics of motivational resilience. Internal dynamics refer to a self-reinforcing cycle wherein the engagement or disaffection of students affects their coping strategies and actions following challenges and setbacks, and external dynamics describe the personal and interpersonal resources and emotional reactivity of students which support or hinder their motivational resilience and then influence their academic achievement (Skinner et al., 2009; Pitzer and Skinner, 2017). According to SSMMD, in classroom settings, specific dimensions of the social context correspond with basic psychological needs (such as competency, autonomy, and relatedness); when the needs of students are satisfied or fulfilled with contextual support, their perceptions of their interactions with teachers shape the self-systems of students (Skinner et al., 2009). As a result, the interpersonal and psychological reciprocal effects of students are related to their academic engagement.

The benefits of student engagement can extend to institutional culture, as Pascarella et al. (2010, p. 21) argue:

*In a dynamic context grounded in an institution’s commitment to improvement, an institutional culture may arise that continuously strives to engage students in effective educational practices and experiences, thereby increasing the likelihood of improved institutional effectiveness and increased student learning and development.*

In this regard, exploring student engagement in disadvantaged areas is necessary.

### Teacher-Student Relationships

The teacher-student relationships lies at the core of schooling experience of students (Pianta and Allen, 2008; Brinkworth et al., 2017). As Noddings (1992) postulates, close teacher-student relationships enable teachers to provide more responsive and sensitive instruction. Relevant research has suggested that teacher-student relationships are formed by continuous interactions between teachers and students, and the relationship includes the meanings of contact with which teachers respond to students (Gehlbach et al., 2016). Specifically, research findings have identified that the accuracy of students in taking perspectives of their teachers and their perceptions of the long-term behavior of their teachers are also crucial factors in responding to changes in or constructing teacher-student relationships (Gehlbach et al., 2012, 2016). This means that the effectiveness of teacher-student interactions that students perceive over a long period of time might be accurate and, in turn, shape their perceptions of teacher-student relationships.

Patterns of teacher-student interactions “depend on the action and reactions of both partners, and their actions and reactions depend on each individual’s perceptions and interpretations of the other’s behavior” (Gable et al., 2003, p. 100). Expectations of teachers on backgrounds of students and evaluations of their behavior or achievements perceived by students might impact student development and the quality of teacher-student relationships (Glock, 2016; Whitaker, 2020). A positive relationship with their teachers predicts improvements in the cooperative and effortful engagement of students in the classroom (Conner and Pope, 2013) and academic achievement (Hamre and Pianta, 2001; Hughes et al., 2008). Studies have verified that when students perceive concerns, encouragement, care, and assistance from the behavior and language of their teachers, their engagement will increase, which in turn contributes to their future academic achievement (Louis and Smith, 1992; Roorda et al., 2011; Gehlbach et al., 2012, 2016; Brinkworth et al., 2017; Lavy and Naama-Ghanayim, 2020). In addition, when teachers were dependable sources of emotional and instrumental support in difficult times, students felt connected to their teacher and safe at school (Furrer et al., 2014), highlighting interpersonal liking and trust with warmth and positive teacher-student relationships. Conversely, students whose relationships with teachers are characterized by conflict are more likely to be held back a grade, to experience peer rejection, and to drop out (Brey and Pauker, 2019; Xuan et al., 2019; Lavy and Naama-Ghanayim, 2020).

Hughes (2011) argues that when children perceive social support in the forms of affection, admiration, satisfaction, and strength of alliances, they develop academically relevant self-views that promote motivated engagement in learning. In line with this perspective, we applied the “student-perceived positive teacher-student relationships” as a research concept to understand how students perceive their relationship with their teachers.

### Self-Worth

The self-worth theory holds that in situations in which poor performance is likely to reveal a low ability, certain (self-worth protective) students intentionally withdraw effort to avoid the negative implications of lower performance in terms of damage to self-worth (Thompson and Dinnel, 2007). According to the definition by Hibbert (2013), self-worth refers to the level of the value and acceptance of being and ability of a person. Individuals tend to devise the rationale underlying their actions and exert effort to construct a reasoning system to interpret their perceived internal and external worlds to identify their being and worth (Li et al., 2020).

Drawing on SSMMD, the most elaborated components of the motivational system are cognitive appraisals, beliefs, and self-perceptions (Skinner et al., 2009). Self-worth, as one of self-perception, is both a source of motivation and psychological vulnerability (Crocker and Knight, 2005). According to the self-worth theory of achievement, motivation, ability, effort, and academic performance are the main elements of self-worth (Covington, 1984), and achievement can be most meaningfully conceptualized in terms of self-perception of causality in return. Source of self-worth of students was often associated with the affirmation of their efforts and with their academic achievement in Chinese education (Zhang and Huang, 1999).

Individuals driven toward academic achievement realize the personal and social benefits of success and gain a reputation for their ability to do so solely through their own efforts (Covington, 1984). A study by Heyman (2008) found that students who exhibit a stable pattern of high academic performance over time may implicitly harbor maladaptive conceptions of ability. Moreover, an experimental finding of seventh-grade students with stereotype threats who are encouraged to view intelligence as malleable demonstrated that academic achievement would increase not due to the attribution of ability (Good et al., 2003). Other construal intervention results also revealed that students from disadvantaged backgrounds have meaningful experiences by strengthening their understanding of the “self” of an individual from the environment and shaping each other instead of attributes of the internal ability, in turn increasing academic performance (Dittmann and Stephens, 2017). Adolescents perceive ability and effort as psychologically equivalent, but effort, not ability, yields a double benefit for the sense of worth of an individual, as being able and virtuous in the view of others (Covington, 1984). If adolescents could perceive positive signs about efforts from the interaction with teachers, the incremental beliefs of self-worth about effort would positively predict student academic achievement (Tarbetsky et al., 2016).

### Teacher-Student Relationships, Self-Worth, and Student Engagement

A supportive teacher-student relationship may provide students with a sense of security that promotes their free and active participation in classroom learning activities. Based on SSMMD, when the needs of students are satisfied or fulfilled with contextual support, their perceptions of their interactions with teachers shape their self-perception, and these interpersonal and psychological reciprocal effects predict the engagement or disaffection of students (Skinner and Belmont, 1993; Pitzer and Skinner, 2017). Studies have identified the alleviation of negative emotions of students (Furrer and Skinner, 2003), feedback of teachers (Wang and Zhang, 2020), and behavior of teachers (Urhahne, 2015) as factors influencing the correlation between teacher-student relationships and student engagement. Positive teacher-student relationships facilitate the effect of external influences on student engagement (Skinner et al., 2008), especially those associated with emotion (Kilday and Ryan, 2019).

Due to the natural cognitive process by which people categorize individuals into social groups depending on their socioeconomic, racial, or cultural background, people view or judge other people as being of the same social group who share important characteristics more similar to what they actually are and may distort perceptions that are even harder to change (Jhangiani and Tarry, 2014). As expected, teachers affect the educational pathways of students directly *via* their evaluation and decision-making of student performance in the classroom life (Timmermans et al., 2016; De Boer et al., 2018; Wang et al., 2018). Student-perceived teacher-student relationships have direct effects on student engagement, forming our first hypothesis (Hypothesis 1, H1).

Self-worth constructed by social interactions can be regarded as a characteristic and an indicator of student engagement (Harter et al., 1998). Studies have investigated the relationship of self-worth to student engagement and/or academic achievement. Wong et al. (2002) identified self-worth as a significant predictor of motivational orientation and academic outcomes based on competence motivation theory. Stahlberg et al. (2019) also demonstrated that self-worth would predict the achievement orientation of students. Based on previous studies, our study hypothesizes that student engagement would be directly affected by self-worth (Hypotheses 2, H2).

Moreover, respect and caring of teachers for students are intertwined with forms of teacher and student engagement that give students an image of themselves and a sense of self-worth (Furrer and Skinner, 2003; Pianta and Allen, 2008; Lavy and Naama-Ghanayim, 2020). When self-worth is based on the perception of an individual on the perception and self-evaluation of social objects, students with high self-worth are more stable and receive higher evaluations and *vice versa* (Zhang and Huang, 1999; Horberg and Chen, 2010; Ryan et al., 2013). Self-worth is highly determined by the perceptions of individuals on the view of the outside world, influenced by positive and negative events, and fluctuates based on the perceptions of students on teacher behavior and language. If self-worth could be a predictor of student engagement and be related to teacher-student relationships, it might have a mediating effect on the relationship with teachers, formulating our third hypothesis (Hypothesis 3, H3).

In addition, studies have confirmed that students with low self-worth are often more sensitive or easily hurt and more awkward in social relationships (Pelham and Swann, 1989), and individuals who lose support from significant others tend to have lower self-worth (Miller, 2000). Crocker and Wolfe (2001) also indicated that greater self-worth could have a positive impact on student learning; however, lower self-worth can contribute to lower school engagement and reduced performance. Thus, self-worth might serve as a moderator in the connection between teacher-student relationships and student engagement, forming the fourth hypothesis of our study (Hypothesis 4, H4).

**FIGURE 1:**
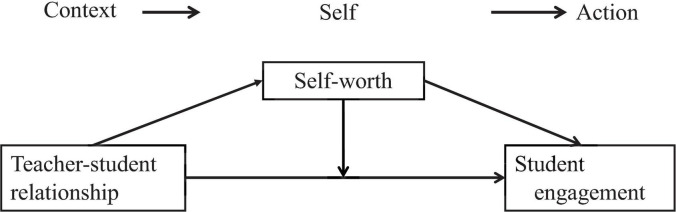
Research model.

Relationships between the variables according to the hypotheses are displayed in Figure 1.

## Materials and Methods

### Participants and Procedure

This present research was a cross-sectional study using quantitative measures conducted in April–May 2018. Considering research findings indicating middle school as a significant period for dropping out in rural China (Yi et al., 2012; Shi et al., 2015), that the self-worth of students decreased among eighth graders in China (Zhang, 1997), that teacher-student relationships decreased among middle school students (Gehlbach et al., 2012), and that the engagement of students decreased during middle school and was particularly severe for students of low socioeconomic, minority, and immigrant backgrounds (Skinner et al., 2009), eighth graders were recruited as targeted participants in rural areas of China.

Interval sampling (e.g., class no. 1, 3, 5, …) was used to select almost half of the eighth-grade classes of an ethnic minority autonomous county located in Yunnan Province of southwest China. A total of 943 eighth-grade students were recruited from 13 secondary schools (i.e., 2 junior high divisions of senior secondary schools and 11 junior secondary schools). A total of 105 respondents with more than 11% missing data were excluded, and 838 (89%) respondents were included in the analysis. According to the exact fit of the confirmatory factor analysis (CFA) [root mean square error of approximation (RMSEA) = 0.06, comparative fit index (CFI) = 0.90] and the minimum power of 80% (π = 0.80; MacCallum et al., 1996), 191 participants were required to estimate the sample sizes of *df*_87_, indicating that the 838 respondent sample of this study was adequate to detect an effect.

Nearly, 45% of respondents were female, 86.1% of the participants were from ethnic minority backgrounds, and approximately, 13% were Han Chinese (missing = 0.8%). Approximately, 90.7% of the participants reported that they lived in rural (township) households, whereas 9.3% of the participants lived in urban (county) households,^1^ revealing the distribution of the type of family and sociodemographic traits in the rural area (Table 1).

**TABLE 1:** Characteristics of respondents.

Variable	Option	Number (%)
Gender	Male	461 (55)
	Female	377 (45)
Ethnicity	Han Chinese	116 (13)
	Ethnic minority	722 (86.1)
Household	Rural	760 (90.7)
	Urban	78 (9.3)

Students were invited to rate their academic performances as being low, medium, or high, and they completed questionnaires about their background (including gender, ethnic minority background, households, and academic performances), positive teacher-student relationships, self-worth, and student engagement. Data collection was conducted by classroom teachers. We provided a detailed protocol to ensure that the questionnaire was administered reliably. Specifically, we provided the same instructions to the survey teachers and asked them to read the instructions out loud to all participants.

Written informed consent, approved by the local Ethics Committee at Beijing Normal University, was obtained from participants and their parents. All participants and parents were informed of the purpose of the study, ensuring that all data would be kept confidential and used only by the research group. Participants were informed that the survey of their perceptions, feelings, and thoughts was voluntary, and they completed a paper-and-pencil questionnaire in 25–30 min.

### Measures

Based on the procedure of direct and reverse translation, the Chinese version of self-report questionnaires was used for data collection. The final version was verified by five experts after three rounds of discussion. All scales were 5-point Likert scales (from 1 = totally disagree to 5 = totally agree), and higher scores represented higher levels of the variable. The items of the measures are presented in the Appendix.

#### Positive Teacher-Student Relationships

The positive teacher-student relationships scale contained six items measuring the perceived relationships of students with their teachers. The effects of positive or negative teacher-student relationships are opposite and are usually offset when summed up, which makes statistical scores unsatisfactory (Liu, 2015). To avoid offsetting the effect, this study modified the scales (Cronbach’s α value ranging between 0.73 and 0.86) of Brinkworth et al. (2017) and adopted positive wording for all items, thereby resulting in a “positive teacher-student relationships” scale such as support, intimacy, and warmth of teachers (e.g., *The interaction with the teacher makes me feel confident and accomplished*; and *the relationships between the teacher and me is close and warm*). Factor loadings ranged from 0.61 to 0.78, and factor loadings ranged between 0.58 and 0.70; construct reliability (CR) = 0.87 and average variance extracted = 0.52. The overall Cronbach’s α value of the scale was 0.86 (>0.70), and the omega value was 0.87. The CFA included χ^2^/(9) = 16.884 (*p* < 0.001), CFI = 0.93, Tucker-Lewis Index (TLI) = 0.93, RMSEA = 0.10, and standardized root mean square residual (SRMR) = 0.05.

#### Self-Worth

The self-worth scale (Cronbach’s α value = 0.81) developed by Crocker et al. (2003) was utilized to measure the perception of students on their self-worth (e.g., *I feel that I am a valuable person, at least at the same level as others; generally, I am satisfied with myself*). The scale included 5 items, and its factor loadings ranged from 0.41 to 0.79, CR = 0.72, and AVE = 0.34. The overall Cronbach’s α value was 0.71, and the omega value was 0.71. The confirmatory factor analyses were χ^2^/(5) = 6.64 (*p* < 0.001), CFI = 0.96, TLI = 0.96, RMSEA = 0.08, and SRMR = 0.04.

#### Student Engagement

The 15-item student engagement scale was developed based on a three-factor instrument (i.e., cognitive, affective, and behavior engagement) (Cronbach’s α value ranging between 0.75 and 0.83) of Fredricks et al. (2005). The fourth factor (i.e., agentic engagement) was developed by the authors using the definition from Reeve (2012), Klemenčič (2017), and Sökmen (2021). Its factor loadings ranged between 0.56 and 0.69, and the exploratory factor analysis (EFA) indicated that student engagement could be separated into four factors with the following loadings, namely, behavioral engagement: 0.70–0.76 (four items, e.g., *I will consult my classmates or teachers if I encounter problems*), cognitive engagement: 0.74–0.82 (three items, e.g., *I will try to connect what I have learned with my own experience*), affective engagement: 0.63–0.74 (five items, e.g., *Learning in class always makes me find it interesting)*, and agentic engagement: 0.70–0.79 (three items, *I will adjust my learning status to keep myself efficient and learn more*) with CR = 0.86 and AVE = 0.60. The overall Cronbach’s α value of the scale was 0.91, and the omega value was 0.86. The CFA results were χ^2^/(59) = 3.194 (*p* < 0.001), CFI = 0.97, TLI = 0.96, RMSEA = 0.05, and SRMR = 0.03.

### Data Analysis

We conducted a series of data analyses. First, statistical descriptions, EFA, and CFA were estimated through composite reliability and convergent validity using the IBM SPSS Amos 22 software. Second, the structural equation modeling (SEM) with latent variables was used to analyze the direct effort of the variables. Since an excessive sample size may have caused the increased Chi-square values (Kline, 2010), a χ^2^/*df* value of five or less is indicative of a good model fit (Kremelberg, 2009). Accordingly, other fit indices were also used to determine how well the model fit, and the model fits well when CFI > 0.90, TLI > 0.90, SRMR < 0.08, and RMSEA < 0.06 (Hu and Bentler, 1999). Third, bootstrapping and Sobel tests were used as estimators for testing mediation effects; zero was not included in the 95% confidence interval for unstandardized and standardized estimates, and the *Z* value was greater than 1.96 and statistically significant at the 0.05 level (Sobel, 1982; Baron and Kenny, 1986). Finally, the multigroup analysis (MGA) with the Amos 22.0 software was conducted to investigate the grouping effect for the moderator of self-worth in the relation of positive teacher-student relationships to student engagement. The participants were grouped into high (i.e., one standard deviation above the mean self-worth score) and low self-worth groups (i.e., one standard deviation under the mean score) to enable clear differentiation for verifying our hypothesis. For each model, confidence intervals for the conditional indirect effects (i.e., at +1 SD or −1 SD of the moderator) were generated with the simple effect analysis.

## Results

### Descriptive Statistics

Prior to the assessment of the hypotheses, descriptive statistics were conducted. The normality assumption (see Appendix) was checked through the values of skewness −1.10 to 0.18 (within −2 and +2) and kurtosis −0.79 to 1.27 (within −7 and +7), indicating that all variables approximating a normal distribution could be accepted (Kline, 2010). The groups of high and low self-worth participants comprised similar numbers of participants and were clearly differentiated. The distribution and Q–Q plots revealed a close to normal distribution of self-worth scores, with mean, median, and mode scores of 17, 17, and 16, respectively. Ultimately, the study classified 130 students into the high self-worth group and 118 students into the low self-worth group.

The study employed different genders and household types as control variables to calculate academic performance and used the mean and standard deviation of the dependent variable positive teacher-student relationships, self-worth, and student engagement. Students in the high self-worth group exhibited substantially higher perceived academic performance, positive teacher-student relationships, and student engagement than those in the low self-worth group. Male students in the high self-worth group had the highest perceived academic performance, whereas male students in the low self-worth group had the poorest self-assessed academic performance (see Table 2).

**TABLE 2:** Descriptive statistics for control and dependent variables among high and low self-worth.

			Academic performance		Teacher-student relationship		Self-worth		Student engagement	
			*M*	SD	*M*	SD	*M*	SD	*M*	SD
High self-worth	Male	Rural	2.95	1.07	22.69	5.70	22.49	1.42	54.07	9.47
		Urban	4.00	1.41	20.50	3.54	21.00	0.00	53.00	9.90
	Female	Rural	3.34	0.99	22.60	6.55	22.19	1.19	55.35	8.67
		Urban	2.60	1.14	22.80	4.44	22.00	1.00	55.00	8.06
Low self-worth	Male	Rural	2.52	1.15	14.81	5.46	11.02	2.18	37.06	10.41
		Urban	2.60	1.67	12.00	2.55	11.20	2.49	38.40	6.35
	Female	Rural	2.94	1.18	18.40	5.84	10.83	2.90	45.54	12.36
		Urban	3.50	0.71	19.00	4.24	10.50	2.12	42.50	4.95

### Correlation Analysis

Table 3 shows that there was a medium (>0.30) positive coefficient between self-worth and positive teacher-student relationships at the level of *p* < 0.001, as well as a significant and moderate positive correlation between self-worth and student engagement and a strong positive correlation between positive teacher-student relationships and student engagement. The findings justified the inclusion of these variables in SEM and the MGA.

**TABLE 3:** Correlations among variables.

	1	2	3
1. Self-worth	–		
2. Positive teacher-student relationship	0.34***	–	
3. Student engagement	0.43***	0.66***	–

***p < 0.001.

## Structural Equation Modeling for Testing Mediation Effects

**FIGURE 2:**
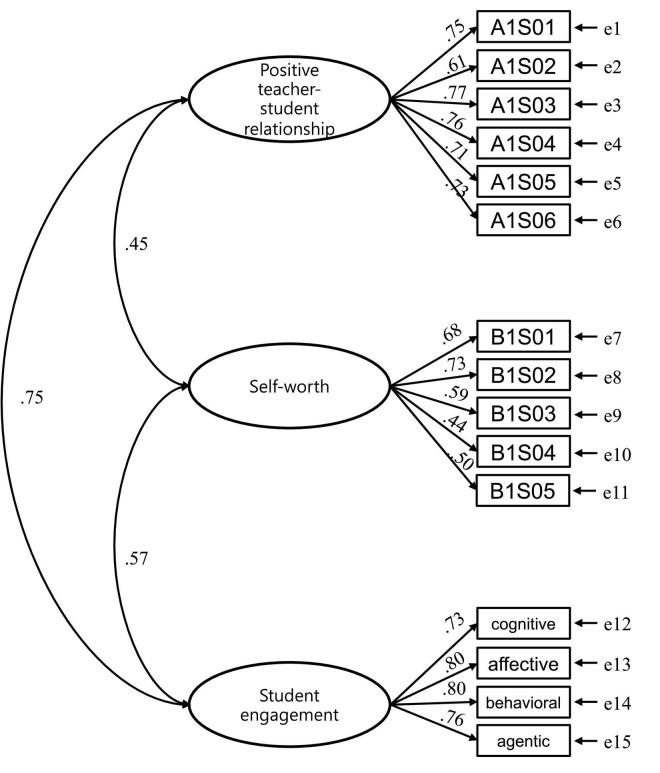
Measurement model.

A measurement model (Figure 2) was examined prior to the model of structural equations. Table 4 illustrates the Chi-square fit statistics/degree of freedom (CMIN/DF) (χ^2^/*df*) ratio for the CFA of the whole measure model. The overall performance of the model satisfactorily met the requirements compared with the baseline parameters, achieving a good fits model.

**TABLE 4:** Confirmatory factor analysis (CFA) indices.

Model fit indices	Achieved values	Baseline values	Remarks
χ^2^	340.17	–	
*df*	87	–	
χ^2^/*df*	3.910	<5	Good fit
Sig (*p*-value)	0	<0.05	Good fit
GFI	0.946	>0.9	Good fit
AGFI	0.926	>0.9	Good fit
SRMR	0.044	<0.08	Good fit
RMSEA	0.059	<0.06	Good fit
NFI	0.934	>0.9	Good fit
RFI	0.921	>0.9	Good fit
IFI	0.950	>0.9	Good fit
TLI	0.940	>0.9	Good fit
CFI	0.950	>0.9	Good fit
PNFI	0.774	>0.5	Good fit
PGFI	0.686	>0.5	Good fit
PCFI	0.787	>0.5	Good fit

*AGFI, adjusted goodness-of-fit index; CFI, comparative fit index; GFI, goodness-of-fit index; NFI, normed fit index; PCFI, parsimony comparative fit index; PNFI, parsimony normed fit index; RFI, relative fit index; RMSEA, root mean square error of approximation; SRMR, standardized root mean square residual; TLI, Tucker-Lewis Index.*

The structural equation modeling was conducted to test the model of positive teacher-student relationships and student engagement directly and self-worth indirectly. All indices displayed a good model fit, χ^2^/*df*(87) = 3.91 (*p* < 0.001), CFI = 0.950 (>0.90), TLI = 0.940 (>0.90), SRMR = 0.046 (<0.08), and RMSEA = 0.059 (<0.06). Loadings of the 15 observed indicators on the relevant latent construct were all as predicted, and all loadings were statistically significant (*p* < 0.001).

**FIGURE 3:**
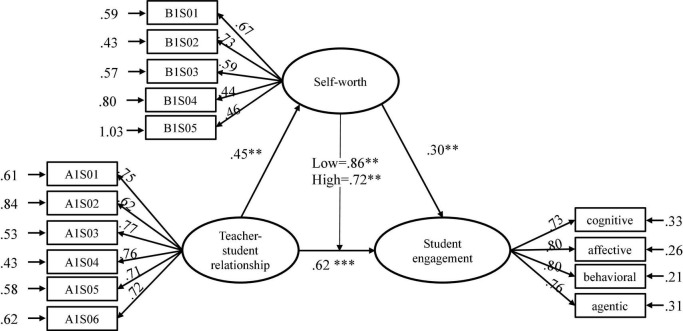
Structural equation modeling (SEM) with path coefficients. **p < 0.01; ***p < 0.001.

As expected, the significant path coefficients (see Figure 3) suggested that both positive teacher-student relationships (β = 0.62, *p* < 0.001) and self-worth (β = 0.30, *p* < 0.01) were significantly positively associated with student engagement, exhibiting a stronger link with student engagement, and positive teacher-student relationships had a stronger impact than self-worth. The results supported the first and second hypotheses (H1 and H2). The direct relation of positive teacher-student relationships to student engagement was statistically significant and remained statistically significant even after controlling for the indirect effect mediated through self-worth, informing the partial mediation effect of self-worth.

The Bootstrap analysis was employed to test the statistical significance of the indirect effects (Shrout and Bolger, 2002). The results showed that the indirect effects of positive teacher-student relationships on student engagement through self-worth were significant by the Sobel test (*Z* = 5.95 > | 1.96|, *p* < 0.001) (Table 5). The 95% confidence intervals through bias correction of unstandardized and standardized coefficients of the indirect effect estimate were 0.58–0.132 (*p* = 0.014) and 0.86–1.87 (*p* = 0.014); zero was not within the intervals, thus, supporting the mediation hypotheses (H3). The findings reported that an important component of student engagement experienced by individuals with positive teacher-student relationships might be due to their self-worth.

**TABLE 5:** Estimators of structural equation modeling (SEM) for mediator.

Path analysis of relationship	B 95% CI [L, H]	SE	*t*	β 95% CI [L, H]
**Direct effect**				
Teacher-student relationship→self-worth (a)	0.36 [0.27, 0.44]	0.041	9.717***	0.45 [0.35, 0.53]
Self-worth→student engagement (b)	0.26 [0.17, 0.36]	0.047	7.534***	0.30 [0.54, 0.68]
Teacher-student relationship→student engagement(c′)	0.43 [0.36, 0.50]	0.036	14.08***	0.62 [0.21, 0.38]
**Total effect**				
Teacher-student relationship→student engagement (c)	0.52 [0.45, 0.59]	0.035	12.11**	0.75 [0.69, 0.79]
**Indirect effect**				
a × b	0.09 [0.06, 0.13]	0.025	5.95***§	0.13 [0.09, 0.19]

Using 2,000 Bootstrap samples. The symbol §by Sobel method, Z value. ***p < 0.001.

### Multigroup Analysis for Testing Moderating Effect

The moderating effect of self-worth was conducted to test the relation between teacher-student relationships and student engagement through MGA (Table 6). The structural relation had a path coefficient value of β = 0.86 for the low self-worth group and β = 0.72 for the high self-worth group, both reaching a positive significance level (*p* < 0.01). This was evidence that the path coefficient value for the low self-worth group was stronger than that for the high self-worth group. The path coefficient difference between the low and high self-worth groups reached significance (Δχ^2^ = 5.852 > 3.84, *df* = 1, *p* < 0.05) and exhibited a direct effect moderation model, supporting the existence of a hypothesized moderating effect (H4). This finding suggested that the engagement of students with low self-worth was affected by a positive teacher-student relationship more than in the case of the high self-worth students.

**TABLE 6:** Hypothesized path comparisons among different groups: moderating effect.

					Compared with non-restricted model
Path/group	*B*	SE	*t*	β	Δχ^2^ (*df* = 1)
TSR-SE					5.852*
Low self-worth	0.71	0.11	6.463***	0.86	
High self-worth	0.41	0.07	5.86***	0.72	

B, unstandardized path coefficient; β, standardized path coefficient; SE, standard error; TSR, teacher-student relationship; SE, student engagement. *p < 0.05 and ***p < 0.001.

## Discussion

First, the findings of the conceptual model test showed that positive teacher-student relationships could predict student engagement (β = 0.62, *p* < 0.001; H1). This result was consistent with other findings (e.g., Zhang and Huang, 1999; Zheng et al., 2004; Roorda et al., 2011; Gehlbach et al., 2012; Brinkworth et al., 2017; Quin, 2017; Kilday and Ryan, 2019; Martin and Collie, 2019). Second, this study applied self-worth as the independent variable, and the finding showed that self-worth had a direct impact on student engagement (β = 0.30, *p* < 0.01; H2), which is in line with previous results that found self-worth to have a significant impact on engagement (Wong et al., 2002; Crocker et al., 2003; Stahlberg et al., 2019).

However, our findings further indicated that the influence effect of positive teacher-student relationships on student engagement was stronger than that of self-worth of students. These findings align with other results that link positive teacher-student relationships with student engagement among students with lower socioeconomic status (e.g., Skinner et al., 2009; Roorda et al., 2011). Students with collective representation and shared meaning from being geographically disadvantaged might make them value themselves less, but this finding reported that the disadvantage would be eliminated through better quality of teacher-student relationships. More importantly, the greater influence of positive teacher-student relationships on student engagement compared with self-worth highlights the importance of interpersonal relationships for rural student engagement. Empirical research has verified that expectations of teachers (e.g., Pianta and Allen, 2008; Timmermans et al., 2016; Hornstra et al., 2018; Wang et al., 2018), attention and behavioral and verbal feedback of teachers, and non-verbal communication impact the quality of teacher-student relationships (Jiang et al., 2018; Brey and Pauker, 2019). Furthermore, social perspective taking of teachers—as they provide a sense of relatedness and help students internalize important academic standards (Verkuyten et al., 2019)—is more important than own judgments of students of their engagement. Consistent with SDT and SSMMD, positive feedback of rural teachers is easier or more related to satisfying the needs of students.

Third, the findings revealed that self-worth partially mediated (i.e., H3) the relation between teacher-student relationships and student engagement, in line with some studies (e.g., Louis and Smith, 1992; Furrer and Skinner, 2003; Pianta and Allen, 2008). The study by Liu et al. (2015) revealed that students with socioeconomic disadvantages in rural China were more responsive and more desiring of positive teacher-student relationships. Another research has indicated a mediating role of student emotions in the relation between teacher-student relationships and student engagement (e.g., Kilday and Ryan, 2019; Chen et al., 2020); notably, this study demonstrated that self-worth could be interpreted as a concrete emotion with a mediating effect. An individual evaluating the self in certain relationships is critical to his or her sense of global worth as a person and as a unique individual (Harter et al., 1998; Ryan et al., 2013; Lavy and Naama-Ghanayim, 2020). Even teachers suppress their stereotypical expectations in regard to judgments about students (Glock and Krolak-Schwerdt, 2014; Glock, 2016). Self-worth has been identified as the individual regarding, loving, and accepting themselves, and feelings of self-worth tend to rise among those who are positively perceived by others and decrease among those who are negatively perceived (Brown and Brown, 2011).

However, our findings further reported that the effects of self-worth might be an interfering factor between teacher-student relationships and student engagement, especially in students with low self-worth (i.e., H4). Although Wu et al. (2010) reported that the positive view of students on teacher-student relationships was associated with concurrent academic competence and general self-worth but not with future engagement, the findings indicated that students with low self-worth are more easily affected by contextual factors than students with high self-worth for future engagement. The moderating role of self-worth should not be neglected. Specifically, this kind of social perspective taking of disadvantaged rural students engenders more negative self-evaluations (Xuan et al., 2019). Such ingrained stereotypes contribute to negative perceptions of academic performance (Crocker and Wolfe, 2001; Timmermans et al., 2016; De Boer et al., 2018; Wang et al., 2018). Students with low self-worth tend to have a less stable self-concept and are more likely to exhibit emotional problems and be affected by external contextual conditions. Supportive teacher-student relationships may contribute to students with low self-worth and stimulate their intrinsic motivation relatedness with positive praise and affirmation.

Self-worth is very important for eighth graders who are facing the developmental task of forming their self-identity and constructing role integration (Ryan et al., 2013), and positive teacher-student relationships are a crucial source of need satisfaction for adolescents (Gehlbach et al., 2012; Jang et al., 2016). Once their needs are satisfied, students are more likely to commit themselves to learning activities and decrease the risk of dropping out (Skinner and Belmont, 1993; Martin and Dowson, 2009; Hornstra et al., 2018).

Moreover, the self-worth of rural students may serve as a protective factor for leading students to perceive positive teacher-student relationships to maintain a reputation as a person of ability who makes an effort, to believe that intelligence is malleable, or to value themselves to improve student engagement. Therefore, facilitating student engagement could reduce or counteract the negative influence of sociogeographic disadvantages (Sinclair et al., 2003), whereas negative teacher-student relationships may cause maladaptive behaviors that would predict adult criminality and alcohol abuse (Wu et al., 2010). The findings of this study implied that the mediating and moderating effects of self-worth should be emphasized more among the association of perceived teacher-student relationships and student engagement. However, the question of how teachers promote student engagement through teacher-student interactions and relationships during middle school is more complex than can be addressed by merely emphasizing the analysis of academic performance (Martin and Collie, 2019). In particular, disadvantaged students need teachers to provide them with respect and equal opportunities to engage in learning (e.g., Louis and Smith, 1992; Whitaker, 2020). Holistic respect, care, and concern of teachers for students are closely associated with happiness and preparedness of students for learning.

## Conclusion

This study provided important insights into the relation between self-worth, teacher-student relationships, and student engagement. Our findings indicated that gender, household type, and academic performance of students did not significantly affect student engagement. The near-normal distribution of self-worth scores suggests that negative stereotypes associated with disadvantaged communities did not impact the self-worth of all the rural students. Rather, the results highlighted that positive teacher-student relationships and self-worth were causally related to the engagement of rural students, and the effect of the former was greater than that of the latter. More importantly, the findings verified not only the mediating effect of self-worth on the relation between positive teacher-student relationships and student engagement but also the moderating effect of positive teacher-student relationships on student engagement, which was stronger for students with low self-worth.

It might be useful to highlight self-worth as one of the core aspired characteristics of student engagement and to maintain a positive teacher-student relationship in rural schools. Studies may underestimate or ignore the importance of self-worth in the association between positive teacher-student relationships and student engagement. It further suggested that caring, warmth, and support of teachers increased, and students might value themselves more positively and feel better about themselves and their lives.

The limitations of the study are that our sample was recruited from a single region in southwestern China, which makes it impossible to generalize our findings. A more diverse sample is needed for future studies. The study was also limited by its focus on self-worth, positive teacher-student relationships, and student engagement; other variables related to contexts and self-concept that may affect student engagement should be explored in further research. According to the findings, self-worth was verified as moderating the relation of positive teacher-student relationships to student engagement but was easily contingent on the interaction with people and the living context, suggesting the need to conduct a multilinear regression in future studies.

## Data Availability Statement

The raw data supporting the conclusions of this article will be made available by the authors, without undue reservation.

## Ethics Statement

Ethical review and approval was not required for the study on human participants in accordance with the local legislation and institutional requirements. Written informed consent from the participants’ legal guardian/next of kin was not required to participate in this study in accordance with the national legislation and the institutional requirements.

## Author Contributions

JH: literature review and first draft. GS: instrument development, data collection, and second draft. TC: statistics and proof-reading. All authors contributed to the article and approved the submitted version.

## Conflict of Interest

The authors declare that the research was conducted in the absence of any commercial or financial relationships that could be construed as a potential conflict of interest.

## Publisher’s Note

All claims expressed in this article are solely those of the authors and do not necessarily represent those of their affiliated organizations, or those of the publisher, the editors and the reviewers. Any product that may be evaluated in this article, or claim that may be made by its manufacturer, is not guaranteed or endorsed by the publisher.

## Footnotes

1 Household (in Chinese, *hukou*) means registered permanent residence and is classified into two types: rural and urban.

## Appendix

**TABLE A1:** The items of all measures.

Number	Variables/items	*M*	SD	*Skewness*	*Kurtosis*
	**Positive teacher-student relationships**				
A1S01	The relationships between me and the teacher is close and warm	3.17	1.18	−0.38	−0.65
A1S02	I am willing to tell the teacher what I feel	2.65	1.16	0.18	−0.79
A1S03	When I am in trouble, the teacher will help me in time	3.58	1.13	−0.73	−0.13
A1S04	As long as I makes progress, the teacher will praise and encourage me	3.68	1.02	−0.85	0.53
A1S05	The interaction with the teacher makes me feel confident and accomplished	3.63	1.08	−0.73	0.05
A1S06	The teacher will listen carefully to my opinions and suggestions	3.46	1.13	−0.55	−0.31
	**Self-worth**				
B1S01	I feel that I am a valuable person, at least on the same level as others	3.45	1.05	−0.57	−0.06
B1S02	I feel I have many good advantages	3.39	0.96	−0.38	−0.08
B1S03	I can do things well like most people	3.68	0.93	−0.68	0.39
B1S04	I have a clear understanding of my strengths and weaknesses	3.59	0.10	−0.56	0.03
B1S05	Generally, I am satisfied with myself	3.10	1.14	−0.10	−0.76
	**Cognitive engagement**	3.77	0.84	−0.89	1.06
CCS01	I will try to determine the cause of mistakes in my homework	3.64	1.03	−0.88	0.45
CCS02	I will try to connect what I have learned with my own experience	3.67	0.97	−0.71	0.31
CCS03	I will correct the wrong homework	3.99	0.95	−1.10	1.27
	**Affective engagement**	3.50	0.85	−0.55	0.44
CAS01	Learning makes me happy	3.44	1.07	−0.65	−0.11
CAS02	I enjoy learning new things	3.72	0.98	−0.87	0.56
CAS03	Learning in class always makes me find it interesting	3.39	1.05	−0.49	−0.14
CAS04	The content in the class is quite interesting and attractive	3.46	1.03	−0.46	−0.23
CAS05	I always try to participate in learning activities in class	3.52	1.05	−0.64	−0.04
	**Behavioral engagement**	3.84	0.77	−0.71	0.88
CBS01	I will listen carefully to the teacher’s explanation	3.84	0.95	−0.99	1.12
CBS02	I will take notes in class	3.89	0.99	−0.94	0.63
CBS03	I will consciously finish my homework on my own	3.87	0.96	−0.90	0.62
CBS04	I will consult my classmates or teachers if I encounter problems	3.77	1.05	−0.89	0.47
	**Agentic engagement**	3.69	0.86	−0.78	0.58
CAS01	I am willing to provide suggestions that will allow classmates to share their learning experience and thought.	3.56	1.05	−0.77	0.14
CAS02	I will adjust my learning status to keep myself efficient and learn more	3.64	0.99	−0.74	0.34
CAS03	I will try my best to make learning more fun	3.86	1.04	−1.00	0.70

*M, mean; SD, standard deviation.*

## References

[B1] Baron R. Kenny D. (1986). The moderator-mediator variable distinction in social psychological research: conceptual, strategic, and statistical considerations. *J. Pers. Soc. Psychol.* 51 1173–1182. 10.1037/0022-3514.51.6.1173 3806354

[B2] Brey E. Pauker K. (2019). Teachers’ nonverbal behaviors influence children’s stereotypic beliefs. *J. Exp. Psychol.* 188:104671. 10.1016/j.jecp.2019.104671 31476615 PMC6768726

[B3] Brinkworth M. E. McIntyre J. Juraschek A. D. Gehlbach H. (2017). Teacher-student relationships: the positives and negatives of assessing both perspectives. *J. Appl. Dev. Psychol.* 55 24–38.

[B4] Brown J. D. Brown M. A. (2011). Self-reflection and feelings of self-worth: When Rosenberg meets Heisenberg. *J. Exp. Soc. Psychol.* 47 1269–1275. 10.1016/j.jesp.2011.05.019

[B5] Chen G. Zhang J. Chan K. Michaels S. Resnick L. B. Huang X. (2020). The link between student-perceived teacher talk and student enjoyment, anxiety and discursive engagement in the classroom. *Br. Educ. Res. J.* 46 631–652.

[B6] Conner J. O. Pope D. C. (2013). Not just Robo-students: Why full engagement matters and how schools can promote it. *J. Youth Adolesc.* 42 1426–1442. 10.1007/s10964-013-9948-y 23592282

[B7] Covington M. V. (1984). The self-worth theory of achievement motivation: findings and implications. *Elem. Sch. J.* 85 4–20.

[B8] Crocker J. (1999). Social stigma and self-esteem: situational construction of self-worth. *J. Exp. Soc. Psychol.* 35 89–107. 10.1006/jesp.1998.1369

[B9] Crocker J. Knight K. M. (2005). Contingencies of self-worth. *Curr. Dir. Psychol. Sci.* 14 200–203. 10.1111/j.0963-7214.2005.00364.x

[B10] Crocker J. Luhtanen R. K. (2003). Level of self-esteem and contingencies of self-worth: unique effects on academic, social, and financial problems in college students. *Pers. Soc. Psychol. Bull.* 29 701–712. 10.1177/0146167203029006003 15189626

[B11] Crocker J. Luhtanen R. K. Cooper M. L. Bouvrette A. (2003). Contingencies of self-worth in college students: theory and measurement. *J. Pers. Soc. Psychol.* 85 894–908. 10.1037/0022-3514.85.5.894 14599252

[B12] Crocker J. Wolfe C. T. (2001). Contingencies of self-worth. *Psychol. Rev.* 108 593–623. 11488379 10.1037/0033-295x.108.3.593

[B13] De Boer H. Timmermans A. C. Van der Werf M. P. C. (2018). The effects of teacher expectation interventions on teachers’ expectations and student achievement: narrative review and meta-analysis. *Educ. Res. Eval.* 24 180–200. 10.1080/13803611.2018.1550834

[B14] Dittmann A. G. Stephens N. M. (2017). Interventions aimed at closing the social class achievement gap: changing individuals, structures, and construals. *Curr. Opin. Psychol.* 18 111–116. 10.1016/j.copsyc.2017.07.044 28869839

[B15] Fredricks J. A. Blumenfeld P. C. Friedel J. Paris A. (2005). School engagement. *Paper Presented at the 2005 Indicators of Positive Development Conference, Child Trends*, Washington, DC. 10.1111/j.1467-9752.2010.00782.x

[B16] Fredricks J. A. Blumenfeld P. C. Paris A. (2004). School engagement: potential of the concept: state of the evidence. *Rev. Educ. Res.* 74 59–119.

[B17] Furrer C. J. Skinner E. A. (2003). Sense of relatedness as a factor in children’s academic engagement and performance. *J. Educ. Psychol.* 95 148–162. 10.1037/0022-0663.95.1.148

[B18] Furrer C. J. Skinner E. A. Pitzer J. R. (2014). The influence of teacher and peer relationships on students’ classroom engagement and everday motivation resilience. *Natl. Soc. Study Educ.* 113 101–123.

[B19] Gable S. L. Reis H. T. Downey G. (2003). He said, she said: a Quasi-signal detection analysis of daily interactions between close relationship partners. *Psychol. Sci.* 14 100–105. 10.1111/1467-9280.t01-1-01426 12661669

[B20] Gehlbach H. Brinkworth M. E. Harris A. D. (2012). Changes in teacher-student relationship. *Br. J. Educ. Psychol.* 82 690–704. 23025399 10.1111/j.2044-8279.2011.02058.x

[B21] Gehlbach H. Brinkworth M. E. King A. M. Hsu L. M. McIntyre J. Rogers T. (2016). Creating birds of similar feathers: leveraging similarity to improve teacher–student relationships and academic achievement. *J. Educ. Psychol.* 108 342–352. 10.1037/edu0000042

[B22] Glock S. (2016). Does ethnicity matter? The impact of stereotypical expectations on in-service teachers’ judgments of students. *Soc. Psychol. Educ.* 19 493–509.

[B23] Glock S. Krolak-Schwerdt S. (2014). Stereotype activation versus application: how teachers process and judge information about students from ethnic minorities and with low socioeconomic background. *Soc. Psychol. Educ.* 17 589–607.

[B24] Good C. Aronson J. Inzlicht M. (2003). Improving adolescents’ standardized test performance: an intervention to reduce the effects of stereotype threat. *Appl. Dev. Psychol.* 24 645–662.

[B25] Hamre B. K. Pianta R. C. (2001). Early teacher-child relationships and the trajectory of children’s school outcomes through eighth grade. *Child Dev.* 72 625–638. 10.1111/1467-8624.00301 11333089

[B26] Harter S. Waters P. Whitesell N. R. (1998). Relational self-worth: differences in perceived worth as a person across interpersonal contexts and adolescents. *Child Dev.* 69 756–766. 10.1111/j.1467-8624.1998.tb06241.x 9680683

[B27] Heyman G. D. (2008). Talking about success: implications for achievement motivation. *J. Appl. Dev. Psychol.* 29 361–370. 10.1016/j.appdev.2008.06.003 19727420 PMC2605085

[B28] Hibbert C. G. (2013). This is How We Grow: A Psychologist’s Memoir of Loss, Motherhood & Discovering Self-Worth & Joy, One Season at a Time. Flagstaff, AZ: Oracle Folio Books.

[B29] Horberg E. J. Chen S. (2010). Significant others and contingencies of self-worth: activation and consequences of relationship-specific contingencies of self-worth. *J. Pers. Soc. Psychol.* 98 77–91. 10.1037/a0016428 20053033

[B30] Hornstra L. Stroet K. van Eijden E. Goudsblom J. Roskamp C. (2018). Teacher expectation effects on need-supportive teaching, student motivation, and engagement: a self-determination perspective. *Educ. Res. Eval.* 24 324–345. 10.1080/13803611.2018.1550841

[B31] Hu L. T. Bentler P. M. (1999). Cutoff criteria for fit indexes in covariance structure analysis: conventional criteria versus new alternatives. *Struct. Equ. Model. Multidiscip. J.* 6 1–55. 10.1080/10705519909540118

[B32] Hughes J. N. (2011). Longitudinal effects of teacher and student perceptions of teacher-student relationship qualities on academic adjustment. *Elem. Sch. J.* 112 38–60. 10.1086/660686 21984843 PMC3187925

[B33] Hughes J. N. Cao Q. (2018). Trajectories of teacher-student warmth and conflict at the transition to middle school: effects on academic engagement and achievement. *J. Sch. Psychol.* 67 148–162. 10.1016/j.jsp.2017.10.003 29571530 PMC5868433

[B34] Hughes J. N. Luo W. Kwok O. Loyd L. (2008). Teacher-student support, effortful engagement, and achievement: a three-year longitudinal study. *J. Educ. Psychol.* 100 1–14. 10.1037/0022-0663.100.1.1 19578558 PMC2705122

[B35] Jang H. Kim E. J. Reeve J. (2016). Why students become more engaged or more disengaged during the semester: a Self-determination theory dual-process model. *Learn. Instr.* 43 27–38. 10.1016/j.learninstruc.2016.01.002

[B36] Jhangiani R. Tarry H. (2014). *Social Categorization and Stereotyping.* Available online at: https://opentextbc.ca/socialpsychology/chapter/social-categorization-and-stereotyping/ (accessed May 27, 2020).

[B37] Jiang R. Liu R.-D. Ding Y. Zhen R. Sun Y. Fu X. (2018). Teacher justice and students’ class identification: belief in a just world and teacher-student relationship as mediators. *Front. Psychol.* 9:802. 10.3389/fpsyg.2018.00802 29875726 PMC5974199

[B38] Khine M. S. (2016). “Non-cognitive skills and factors in educational success and academic achievement,” in *Non-Cognitive Skills and Factors in Educational Attainment*, eds Khine M. S. Areepattamannil S. (Boston, MA: Sense Publishers), 3–9. 10.1007/978-94-6300-591-3_1

[B39] Kilday J. E. Ryan A. M. (2019). Personal and collective perceptions of social support: implications for classroom engagement in early adolescence. *Contemp. Educ. Psychol.* 58 163–174. 10.1016/j.cedpsych.2019.03.006

[B40] Klemenčič M. (2017). From student engagement to student agency: conceptual considerations of European policies on student-centered learning in higher education. *High. Educ. Policy* 30 69–85. 10.1057/s41307-016-0034-4

[B41] Kline R. B. (2010). *Principles and Practice of Structural Equation Modeling*, 2nd Edn. New York, NY: Guilford Press.

[B42] Kremelberg D. (2009). *Practical Statistics.* Thousand Oaks, CA: SAGE Publications Inc.

[B43] Kuh G. D. Kinzie J. Buckley J. A. Bridges B. K. Hayek J. C. (2007). *Piecing Together the Student Success Puzzle: Research, Propositions, and Recommendations*. ASHE Higher Education Report, 32. Hoboken, NJ: John Wiley & Sons, Inc.

[B44] Lavy S. Naama-Ghanayim E. (2020). Why care about caring? Linking teachers’ caring and sense of meaning at work with students’ self-esteem, well-being, and school engagement. *Teach. Teach. Educ.* 91 1–12.

[B45] Lei H. Cui Y. Zhou W. (2018). Relationships between student engagement and academic achievement: a meta-analysis. *Soc. Behav. Pers.* 46 517–528.

[B46] Li D. Guo Y. Zhang L. Tu M. Yu Q. Li H. (2020). Fluid self-worth: the compensatory role of online social interaction. *Child. Youth Serv. Rev.* 119:105536. 10.1016/j.childyouth.2020.105536

[B47] Liu C. H. (2015). The effects of the success and failure contingencies of self-worth on college students’ depression tendencies. *J. Educ. Psychol.* 38 65–91.

[B48] Liu H. X. (2019). Improving middle school students’ self-worth: using practical activities during winter vacation for research and extension of early semester life as an example. *Jiangsu Educ.* 7 62–64.

[B49] Liu Y. Li X. Chen L. Qu Z. (2015). Perceived positive teacher-student relationship as a protective factor for Chinese left-behind children’s emotional and behavioral adjustment. *Int. J. Psychol.* 50 354–362. 10.1002/ijop.12112 25410645

[B50] Louis K. S. Smith B. A. (1992). “Cultivating teacher engagement: breaking the iron law of social class,” in *Student Engagement and Achievement in American Secondary School*, ed. Newmann F. M. (New York, NY: Teachers College Press), 119–152.

[B51] MacCallum R. C. Browne M. W. Sugawara H. N. (1996). Power analysis and determination of sample size for covariance structure modeling. *Psychol. Methods* 1 130–149. 10.1037/1082-989x.1.2.130

[B52] Martin A. J. Collie R. J. (2019). Teacher–student relationships and students’ engagement in high school: Does the number of negative and positive relationships with teachers matter? *J. Educ. Psychol.* 111 861–876.

[B53] Martin A. J. Dowson M. (2009). Interpersonal relationships, motivation, engagement, and achievement: yields for theory, current issues, and educational practice. *Rev. Educ. Res.* 79 327–365. 10.3102/0034654308325583

[B54] Miller H. M. (2000). Cross-cultural validity of a model of self-worth: application to Finnish children. *Soc. Behav. Pers.* 28 105–118.

[B55] Newmann F. M. Wehlage G. G. Lamborn S. D. (1992). “The significance and sources of student engagement,” in *Student Engagement and Achievement in American Secondary Schools*, ed. Newmann F. M. (New York, NY: Teachers College Press), 11–39.

[B56] Noddings N. (1992). *The Challenge to Care in Schools: an Alternative Approach to Education*. New York, NY: Teachers College Press.

[B57] Pan J. Zaff J. F. Donlan A. E. (2017). Social support and academic engagement among reconnected youth: adverse life experiences as a moderator. *J. Res. Adolesc.* 27 890–906. 10.1111/jora.12322 29152870

[B58] Pascarella E. T. Seifert T. A. Blaich C. (2010). How effective are the NSSE benchmarks in predicting important educational outcomes? *Change* 42 16–22.

[B59] Pelham B. W. Swann W. B. Jr. (1989). From self-conceptions to self-worth: on the sources and structure of global self-esteem. *J. Pers. Soc. Psychol.* 57 672–680. 10.1037//0022-3514.57.4.672 2795437

[B60] Pianta R. C. Allen J. P. (2008). “Building capacity for positive youth development in secondary school classrooms: changing teachers’ interactions with students,” in *Toward Positive Youth Development: Transforming Schools and Community Programs*, eds Shinn M. Yoshikawa H. (Oxford: Oxford University Press), 21–39. 10.1093/acprof:oso/9780195327892.003.0002

[B61] Pitzer J. Skinner E. (2017). Predictors of changes in students’ motivational resilience over the school year: the roles of teacher support, self-appraisals, and emotional reactivity. *Int. J. Behav. Dev.* 41 15–29. 10.1177/0165025416642051

[B62] Quin D. (2017). Longitudinal and contextual associations between teacher-student relationships and student engagement: a systematic review. *Rev. Educ. Res.* 87 345–387.

[B63] Reeve J. (2012). “A self-determination theory perspective on student engagement,” in *Handbook of Research on Student Engagement*, eds Christenson S. J. Reschly A. L. Wylie C. (New York, NY: Springer), 149–172. 10.1007/978-1-4614-2018-7_7

[B64] Roorda D. L. Koomen H. M. Spilt J. L. Oort F. J. (2011). The influence of affective teacher-student relationships on students’ learning engagement and achievement: a meta-analytic approach. *Rev. Educ. Res.* 81 493–529. 10.3102/0034654311421793

[B65] Ryan A. M. Shim S. S. Makara K. A. (2013). Changes in academic adjustment and relational self-worth across the transition to middle school. *J. Youth Adolesc.* 42 1372–1384. 10.1007/s10964-013-9984-7 23873280

[B66] Shi Y. J. Zhang L. X. Ma Y. Yi M. M. (2015). Dropping out of rural China’s secondary schools: a mixed-methods analysis. *China Q.* 224 1048–1069. 10.1017/s0305741015001277

[B67] Shrout P. E. Bolger N. (2002). Mediation in experimental and nonexperimental studies: new procedures and recommendations. *Psychol. Methods* 7 422–445. 10.1037/1082-989x.7.4.422 12530702

[B68] Sinclair M. F. Christenson S. L. Lehr C. A. Anderson A. R. (2003). Facilitating student engagement: lessons learned from Check & Connect longitudinal studies. *Calif. School Psychol.* 8 29–41. 10.1111/tct.13437 34786855

[B69] Skinner E. Furrer C. Marchand G. Kindermann T. (2008). Engagement and disaffection in the classroom: Part of a larger motivational dynamic? *J. Educ. Psychol.* 100 765–781. 10.1037/a0012840

[B70] Skinner E. A. Belmont M. J. (1993). Motivation in the classroom: reciprocal effect of teacher behavior and student engagement across the school year. *J. Educ. Psychol.* 85 571–581. 10.1037/0022-0663.85.4.571

[B71] Skinner E. A. Kindermann T. A. Connell J. P. Wellborn J. G. (2009). “Engagement and disaffection as organizational constructs in the dynamics of motivational development,” in *Handbook of Motivation in School*, eds Wentzel K. Wigfield A. (New York, NY: Routledge), 223–245.

[B72] Skinner E. A. Pitzer J. R. (2012). “Developmental dynamics of student engagement, coping, and everyday resilience,” in *Handbook of Research on Student Engagement*, eds Christenson S. L. Reschly A. L. Wylie C. (New York, NY: Springer), 21–44. 10.1007/978-1-4614-2018-7_2

[B73] Sobel M. E. (1982). “Asymptotic confidence intervals for indirect effects in structural equation models,” in *Sociological Methodology*, ed. Leinhardt S. (Washington DC: American Sociological Association), 290–312. 10.2307/270723

[B74] Sökmen Y. (2021). The role of self-efficacy in the relationship between the learning environment and student engagement. *Educ. Stud.* 47 19–37. 10.1080/03055698.2019.1665986

[B75] Stahlberg J. Tuominen H. Pulkka A. Niemivirta M. (2019). Maintaining the self? Exploring the connections between students’ perfectionistic profiles, self-worth contingency, and achievement goal orientations. *Pers. Individ. Dif.* 151:109495.

[B76] Tarbetsky A. L. Collie R. J. Martin A. J. (2016). The role of implicit theories of intelligence and ability in predicting achievement for indigenous Australian students. *Contemp. Educ. Psychol.* 47 61–71. 10.1016/j.cedpsych.2016.01.002

[B77] Thompson T. Dinnel D. L. (2007). Poor Performance in mathematics: Is there a basis for a self-worth explanation for women? *Educ. Psychol.* 27 377–399. 10.1080/01443410601104197

[B78] Timmermans A. C. De Boer H. Van der Werf M. P. C. (2016). An investigation of the relationship between teachers’ expectations and teachers’ perceptions of student attributes. *Soc. Psychol. Educ.* 19 217–240. 10.1007/s11218-015-9326-6

[B79] Urhahne D. (2015). Teacher behavior as a mediator of the relationship between teacher judgment and students’ motivation and emotion. *Teach. Teach. Educ.* 45 73–82. 10.1016/j.tate.2014.09.006

[B80] Verkuyten M. Thijs J. Gharaei N. (2019). Discrimination and academic (dis)engagement of ethnic-racial minority students: A social identity threat perspective. *Soc. Psychol. Educ.* 22 267–290.

[B81] Wang A. Rubie-Davies C. M. Meissel K. (2018). A systematic review of the teacher expectation literature over the past 30 years. *Educ. Res. Eval.* 24 124–179. 10.1080/13803611.2018.1548798

[B82] Wang M. T. Holcombe R. (2010). Adolescents perceptions of school environment, engagement, and academic achievement in middle school. *Am. Educ. Res. J.* 47 633–662. 10.3102/0002831209361209

[B83] Wang S. Zhang D. (2020). Perceived teacher feedback and academic performance: The mediating effect of learning engagement and moderating effect of assessment characteristics. *Assess. Eval. High. Educ.* 45 973–987.

[B84] Whitaker M. C. (2020). Us and them: using social identity theory to explain and re-envision teacher-student relationships in urban schools. *Urban Rev.* 52 691–707. 10.1007/s11256-019-00539-w

[B85] Wong E. H. Wiest D. J. Cusick L. B. (2002). Perceptions of autonomy support, parent attachment, competence and self-worth as predictors of motivational orientation and academic achievement: an examination of sixth- and ninth-grade regular education students. *Adolescence* 37 255–266. 12144158

[B86] Wu J. Y. Hughes J. N. Kwok O. M. (2010). Teacher student relationship quality type in elementary grades: effects on trajectories for achievement and engagement. *J. Sch. Psychol.* 48 357–387. 10.1016/j.jsp.2010.06.004 20728688 PMC2928164

[B87] Xuan X. Xue Y. Zhang C. Luo Y. Jian W. Qi M. (2019). Relationship among school socioeconomic status, teacher-student relationship, and middle school students’ academic achievement in China: using the multilevel mediation model. *PLoS One* 14:e0213783. 10.1371/journal.pone.0213783 30893361 PMC6426256

[B88] Yi H. M. Zhang L. X. Luo R. F. Shi Y. J. Mo D. Chen X. X. (2012). Dropping out: Why are students leaving junior high in China’s poor rural areas? *Int. J. Educ. Dev.* 32 555–563.

[B89] Yue P. Lu Y. Chen L. (2016). Analysis of the reasons for the drop-out of rural students: a case study of a county in Guizhou. *J. Guizhou Educ. Univ.* 32 71–74.

[B90] Zhang W. (1997). A preliminary research on characteristics of middle school students’ self-worth. *Psychol. Sci.* 20:504.

[B91] Zhang Y. Huang X. (1999). The characteristics of achievement motivation of people with low feeling of self-worth. *J. Southwest China Normal Univ.* 25 82–86.

[B92] Zhao G. Ye J. Li Z. Xue S. (2017). How and why do Chinese urban students outperform their rural counterparts? *China Econ. Rev.* 45 103–123.

[B93] Zheng H. Liu X. Mo L. (2004). The relationship between eighth graders perceived teacher-expectancy, self-worth and goal orientation. *Psychol. Dev. Educ.* 3 16–22.

